# Soft Robotic Grippers for Biological Sampling on Deep Reefs

**DOI:** 10.1089/soro.2015.0019

**Published:** 2016-03-01

**Authors:** Kevin C. Galloway, Kaitlyn P. Becker, Brennan Phillips, Jordan Kirby, Stephen Licht, Dan Tchernov, Robert J. Wood, David F. Gruber

**Affiliations:** ^1^ Wyss Institute for Biologically Inspired Engineering, Harvard University, Cambridge, Massachusetts.; ^2^ Harvard John A. Paulson School of Engineering and Applied Sciences, Harvard University, Cambridge, Massachusetts.; ^3^ Department of Ocean Engineering, University of Rhode Island, Narragansett, Rhode Island.; ^4^ Leon Charney School of Marine Sciences, Haifa University, Haifa, Israel.; ^5^ Department of Natural Sciences, Baruch College, City University of New York, New York, New York.; ^6^ American Museum of Natural History, Sackler Institute of Comparative Genomics, New York, New York.

## Abstract

This article presents the development of an underwater gripper that utilizes soft robotics technology to delicately manipulate and sample fragile species on the deep reef. Existing solutions for deep sea robotic manipulation have historically been driven by the oil industry, resulting in destructive interactions with undersea life. Soft material robotics relies on compliant materials that are inherently impedance matched to natural environments and to soft or fragile organisms. We demonstrate design principles for soft robot end effectors, bench-top characterization of their grasping performance, and conclude by describing *in situ* testing at mesophotic depths. The result is the first use of soft robotics in the deep sea for the nondestructive sampling of benthic fauna.

## Introduction

Deep and mesophotic coral reefs, as well as other deep sea ecosystems in general, are hotspots for unique biological diversity and genetic adaptions. Although the existence of coral reefs down to 128 m was noted by Darwin in 1889,^1^ it was not until the recent advent of technical diving, remotely operated vehicles (ROVs) and submersibles that researchers have been able to access and examine them *in situ*. In the few decades since, the field of mesophotic reef biology has expanded significantly with the increased access.^2–5^ Concurrently, the scientific community is presented with estimates that 19% of the world's shallow coral reefs have recently been lost, with a further 35% expected to disappear in the next 40 years.^6^ However, reefs occurring at depths greater than 30 m are somewhat buffered from human and natural disturbances and represent a potential refuge.^7,8^ These, deep reefs remain dramatically under studied compared to other highly diverse habitats.^9,10^

Although major advances have been made in accessing this environment, biological collection and the molecular biology and biochemical analysis of these habitats are still highly challenging. Intervention almost always involves a robotic manipulator, and the industrial nature of existing technology causes a major challenge for researchers valuing delicate collections. Deep reefs are known to have slow growth rates and experience seasonal bleaching events,^11^ so it is of interest for scientists to take steps to study these ecosystems with as great care as possible.

Commercially available deep sea manipulation systems are designed to perform heavy mechanical work (i.e., construction or pipeline maintenance) and are not geared to perform delicate tasks, such as the collection of fragile biological specimens. For example, high-end systems* incorporate sophisticated force feedback to minimize damage from their rigid jaws. These arms can generate lifting and gripping forces up to 500 lbf and are not optimal for delicate specimen collection. Furthermore, more economical grippers^†^ typically lack force feedback and an intuitive user interface. Hence, many biologists work to modify research design and collection methods and tools as best possible to suit their needs and consider getting the sample to the surface, regardless of condition, as a success.

**FIG. 1.**
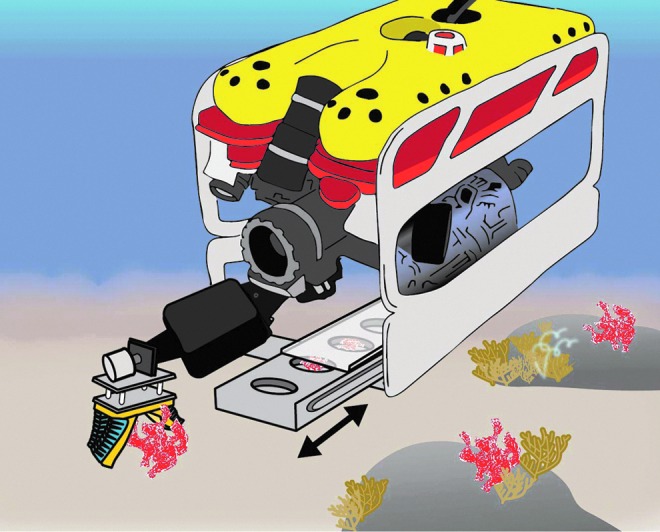
Concept figure of Seaeye Falcon remotely operated vehicle (ROV) with soft robotic manipulator handling an urchin. Color images available online at www.liebertpub.com/soro

Recently, the field of soft robotics has erupted as an alternative to hard-material robotics, providing a safer alternative for robots to interact, in proximity, with living organisms.^12–18^ By using soft materials instead of more traditional metals and hard polymers, robotic components can closely mimic the properties of natural systems. This can allow marine and molecular biologists to delicately handle an organism while in its natural setting (see concept Fig. 1) and perform more complicated tasks while on location underwater.

## Related Work

Underactuated, compliant grippers have proven to be a robust option for manipulating a wide variety of object shapes and sizes in unstructured environments. Recent research from Stanford University explored the development of a compliant, underactuated gripper to augment human capabilities and reduce strain-related injuries for professional divers working at depths up to 100 m. Their gripper mimics the grasps needed to manipulate welding equipment and power tools that would otherwise be used by human hands.^19^ The design and actuation of the compliant underwater gripper demonstrated by Stanford are similar to the shape deposition manufactured hand developed at Harvard, using a single tendon to drive each finger and coupling the rigid polymer phalanges of each finger with elastomeric flexure joints.^20^

Similar to underactuated, compliant hands, soft robotic grippers are adept at adapting to variations in object size and shape. Furthermore, soft robotic systems are ideally suited for gripping and manipulating delicate objects and complex shapes by conforming to the object's shape and distributing grasping forces. Soft systems also offer improved safety as these pneumatically and hydraulically actuated soft materials are inherently safe for interfacing with humans and animals because of their natural compliance and back drivability.^21,22^ Suzumori conducted some the earliest work on soft robotic grippers, in which he created continuum-style soft actuators that consisted of three parallel, fiber-reinforced elastomeric chambers spaced evenly around a central axis. Coordinated fluid pressurization of the actuator's chambers can produce multidegree-of-freedom bending and can be used as fingers to create soft robotic grippers.^23^ In fact, Lane *et al.* designed a soft robotic subsea hand with a version of continuum actuator fingers; however, we were unable to find any historical record that evaluates the gripper on an ROV in open water.^24^

In addition to soft grippers, several soft robotic continuum arms have also been developed for the purpose of grasping. Two such arms, the OCTOPUS robot by Cianchetti *et al.*^13^ and the OCTarm by Walker *et al.*,^25^ have demonstrated grasping in shallow waters, but have not operated at significant depths.

The soft robotic actuators employed in our grippers are monolithic structures (i.e., no moving parts such as pin joints, gears, or linkages) and are modularly coupled to a palm. Their structure is based on the PneuNet and fiber-reinforced soft actuators developed at Harvard^16,21^ and have proven robust at depths greater than 800 m underwater in field testing (see Appendix for further discussion on depth testing). The simple construction, inexpensive materials, and modular design of our soft grippers allow us to quickly modify or repair a system in the field with minimal expense and mechanical expertise.

## Soft Robotic Gripper

### ROV system description

**FIG. 2.**
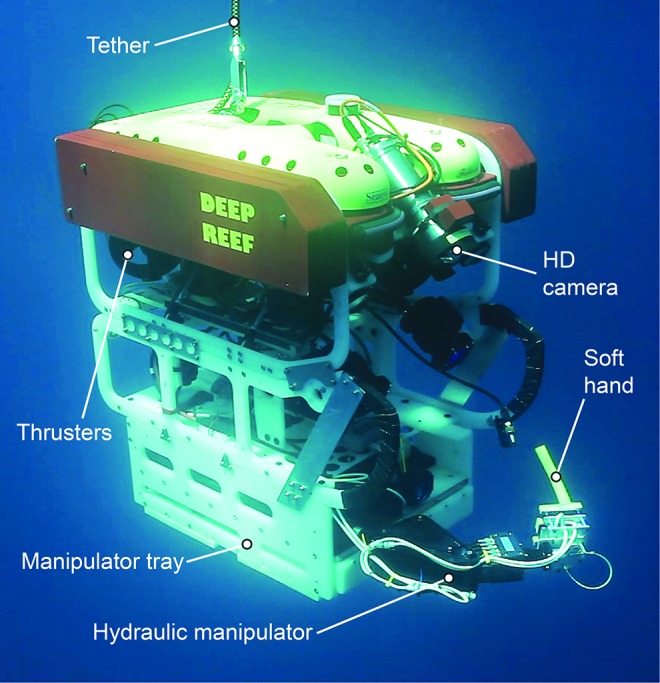
The Seaeye Falcon submersible—aka Deep Reef ROV—was the platform used for all the deep sea soft robotic gripper experiments. Color images available online at www.liebertpub.com/soro

A heavily modified Saab Seaeye Falcon ROV (Fig. 2) was used as a platform for *in situ* testing and field collections. This small research class ROV measures 1 m long ×0.6 m wide ×1 m tall, with a launch weight of 300 kg (660 lbs.) and a 300 m depth rating. Attached to the bottom of the ROV is a Hydro-Lek (HLK-43000)^2^ 5 degrees of freedom (DOF) hydraulic powered manipulator with a 55.3 cm long arm and a lift capacity of 20 kg (44 lbs.) at full reach in air. Two of the arm functions include a 180° wrist rotation and a linear push/pull piston with ∼2.5 cm (1 inch) of travel. The latter function controls the opening and closing of a metal gripper that is clamped to the wrist DOF. The arm is operated through an open loop control box where the operator controls each DOF with a panel of toggle switches and relies on live video feed from an onboard high definition camera to coordinate arm movements.

### Hydraulic system

**FIG. 3.**
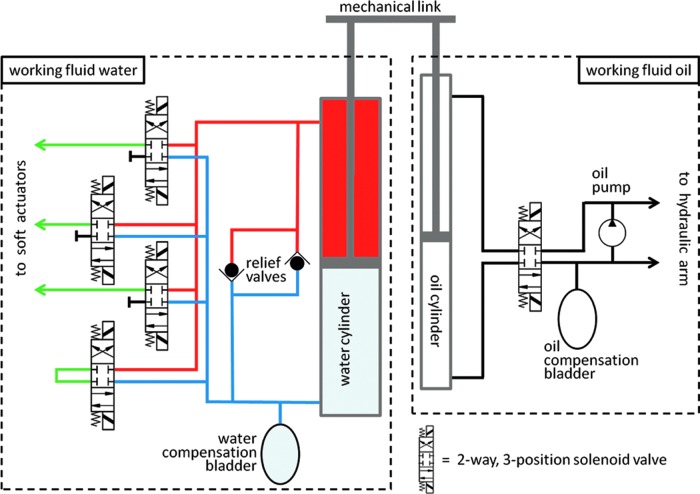
Schematic of the hydraulic system used to power the soft robotic gripper. Color images available online at www.liebertpub.com/soro

Water flow into and out of the fingers was controlled by a system consisting of a pair of double-acting cylinders and four closed-center, three-way, two-position solenoid valves (see schematic in Fig. 3). The piston rods of the water- and oil-filled cylinders were mechanically linked. Using the control box, the operator could activate the solenoid valves (a modified Hydro-Lek HLK7020 valve pack) to connect the water-filled cylinder to any combination of three outputs to the soft actuators. The direction of travel of the oil-filled cylinder was then chosen to apply either pressure or suction to the selected fingers.

The oil-filled piston was driven at a working pressure of 1500 psi by the same pump used to drive the hydraulic arm. Given minimal backpressure from the fingers compared to the rated load, the cylinder speed was determined by the internal flow resistance of the hydraulic circuit. As a result, the linked cylinders functioned as a bidirectional, near-constant flow source, with the output flow rate determined by the area ratio between the input and output cylinders. The dual piston approach also ensured that the finger working fluid and the arm hydraulic fluid were isolated from one another.

The second side of the water-filled cylinder was connected to a flexible bladder exposed to seawater. Pressure within the fingers, and throughout the system, could thus be equalized to ambient pressure through a loop-back valve connecting the two water cylinder chambers. Finally, a two-way safety manifold allowed selection of maximum and minimum pressures in the fingers, with respect to ambient pressure.

### Soft gripper design requirements

Given the ROV and manipulator arm platform, the primary goal of this project was to replace the factory-furnished metal gripper with a soft robotic hand and demonstrate the advantages of soft systems for deep coral manipulation and sampling. The functional requirements for the soft gripper design can be broadly categorized under two development topics: integration and operation. With respect to integration, the soft gripper hardware had to connect to the existing Hydro-Lek arm, use the ROV's hydraulic system to drive the soft actuators, and to use the arm's linear push/pull piston to control a cutting mechanism. With respect to operation, the soft gripper had to be versatile by supporting the quick soft actuator installation, adjustment (e.g., orientation and spacing), and removal. Furthermore, we favored designs that supported the addition and removal of textures and other accessories (e.g., netting material bridging the space between neighboring actuators) so that the capabilities of the actuators could be modified in the field. Lastly, the actuators had to survive and operate at mesophotic depths (>300 m).

To test the effects of high hydrostatic pressure on soft actuators, two fiber-reinforced soft actuators were hydraulically pressurized and deployed in a static configuration at a depth of ∼800 m. Video was recorded to ensure the grippers maintained position and structural integrity during descent and ascent.

### Soft robotic gripper design

**FIG. 4.**
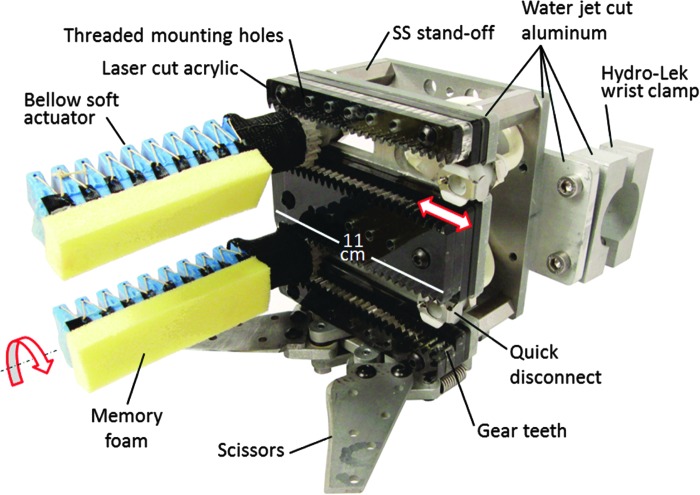
Assembled soft robotic gripper featuring two bellows-style soft actuators with memory foam textures. The *rectangular* shaped palm measures 11 × 10 cm. SS, stainless steel. Color images available online at www.liebertpub.com/soro

The soft gripper's palm is designed for rapid and inexpensive customization. The palm structure was assembled from sheet materials: water jet-cut aluminum and laser-cut acrylic (Fig. 4). Four thru-wall female quick-disconnect fittings were positioned in recessed channels formed from stacked layers of acrylic. The relative spacing of the fittings can be manually adjusted. Furthermore, laser-cut gear teeth in the acrylic run parallel to the slots and match the 3D-printed spur gear teeth integrated into the soft actuator base. When the gear teeth engage, this locks the position of the female quick-disconnect fitting and orientation of the soft actuator. Note that without the gear teeth, the actuator can passively rotate about the axis of the quick-disconnect fitting. The palm surface also features a variety of threaded holes that serve as anchor points for palm textures and other accessories. Stainless steel stand-offs create an open cavity between two aluminum plates for routing hydraulic plumbing to the female quick disconnects. These plates also serve as anchor points for a Bowden cable-driven, four-bar linkage cutting mechanism. Custom fittings were machined to connect the Hydro-Lek arm's push-pull rod to the cutter. Vented screws terminate each end of the Bowden cable sheath and were tightened or loosened to tension the cable. Cutting blades were screw mounted to one of the linkages, enabling replacement with and evaluation of new blade designs.

## Soft Actuator Development

Early in the design of the soft gripper, we identified desirable performance criteria for the soft actuators, which included the ability to distribute forces over a large area, conform to irregular shapes, and the ability to alter the surface texture of the actuator. Two different soft actuator architectures—fiber reinforced and bellows type—were chosen that led to innovations in fabrication and materials integration.

### Boa-type fiber-reinforced soft actuator

**FIG. 5.**
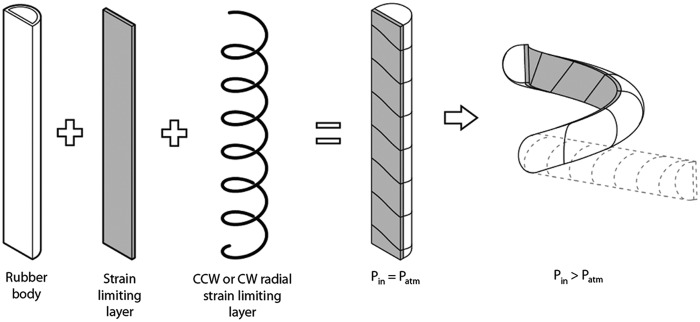
Components of a fiber-reinforced boa-type actuator (adapted from Polygerinos *et al.*^26^).

In previous work,^26^ we presented a multistep soft actuator manufacturing method for molding elastomeric tubular bladders, whereby fiber reinforcements can be embedded in the actuator wall to program the material's strain response to a pressurized fluid input. With this technique, soft actuators can be designed to execute a variety of complex motions from a single pressurized fluid source. For example, the boa-type actuator (Fig. 5) has a fiber-reinforcement strategy that causes the elastomeric structure to simultaneously bend and twist to form a coil.

**FIG. 6.**
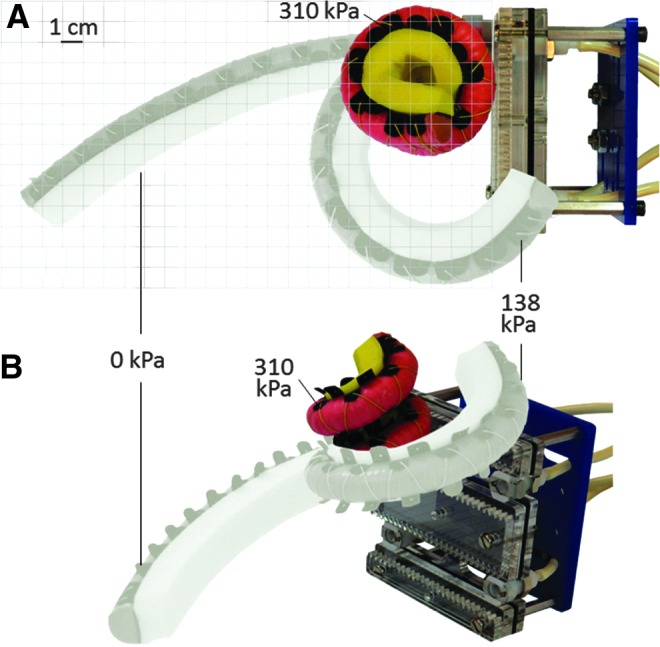
**(A)** Top view of the boa-type actuator's range of motion against an approximated grid scale. **(B)** Isometric view of the actuator's range of motion. Color images available online at www.liebertpub.com/soro

Inspired by the boa constrictor and tentacled cephalopods, the boa-type soft actuator can access tight spaces and reversibly shape change from a nearly straight beam to a helical structure (Fig. 6). This simple motion path, which is dependent on the actuator geometry, elastomeric material properties, and fiber-reinforcement placement, gives the actuator the unique ability to tolerate uncertainty of sample size, shape, and stiffness. As interior fluid pressure increases, the 300 mm long, narrow (approximately a 15 mm half-round diameter) actuator wraps around an object to increase surface area contact and distribute forces. The boa-type actuator we developed for field testing can wrap around objects as small as 12 mm in diameter.

### Fabrication of a boa-type fiber-reinforced soft actuator

**FIG. 7.**
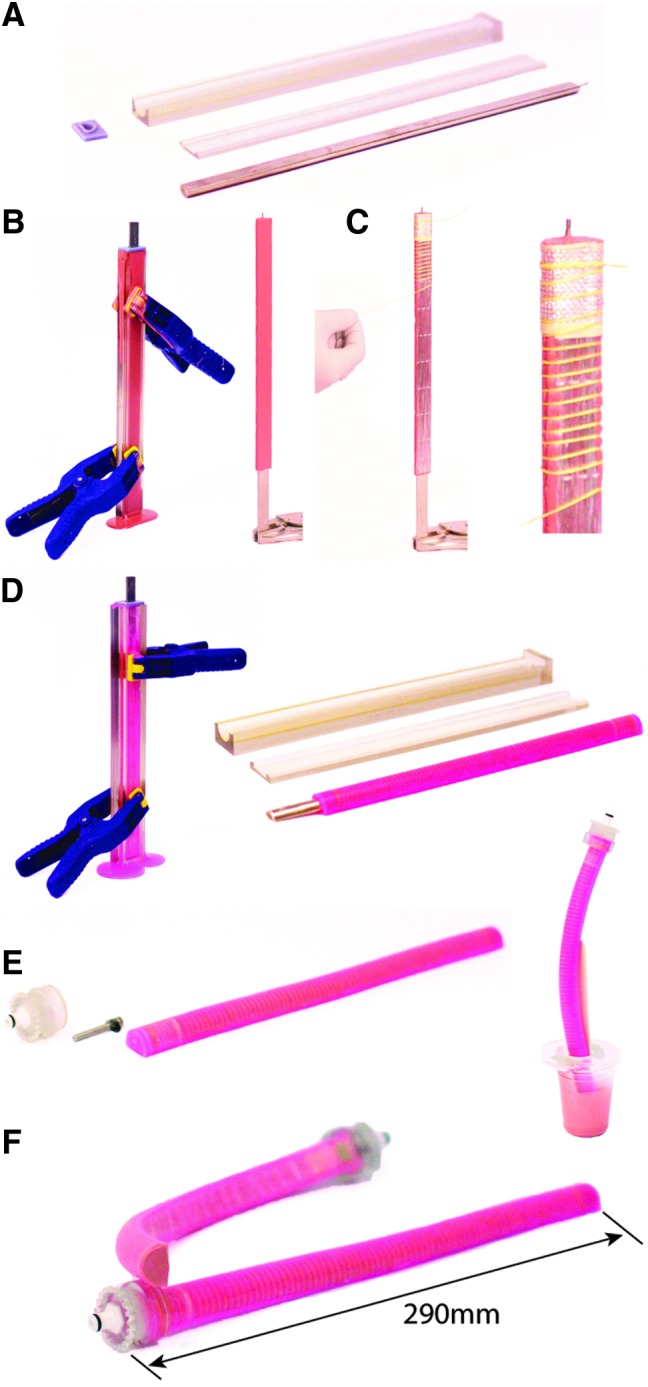
Schematic diagram outlining the stages of the boa-type actuator fabrication process. **(A)** The actuator bladder is molded using 3D-printed molds and the internal geometry is formed with a half-round steel rod. **(B)** Liquid polymer (M4601 by Wacker Chemical, Inc.) is poured into the clamped mold and the half-round rod is inserted into the center. The polymer is cured and removed from the mold. **(C)** Strain-limiting materials (i.e., fiber reinforcements) are applied to the exterior of the bladder. **(D)** The fiber-reinforced bladder is inserted into a second mold filled with liquid polymer (Dragon Skin 20 by Smooth On) to add a thin skin (∼1 mm thick) around the actuator body to hold the strain-limiting materials in place. The actuator body is then removed from the mold. **(E)** The half-round rod is removed and coupling hardware is installed on one end of the actuator. The other end of the actuator is sealed with more polymer. **(F)** Excess polymer is trimmed from the end and the actuator is complete. Color images available online at www.liebertpub.com/soro

The boa-type actuator fabrication process has been recorded in detail in Figure 7, with a written description of each step given in the caption.

### Bellows-type soft actuator

**FIG. 8.**
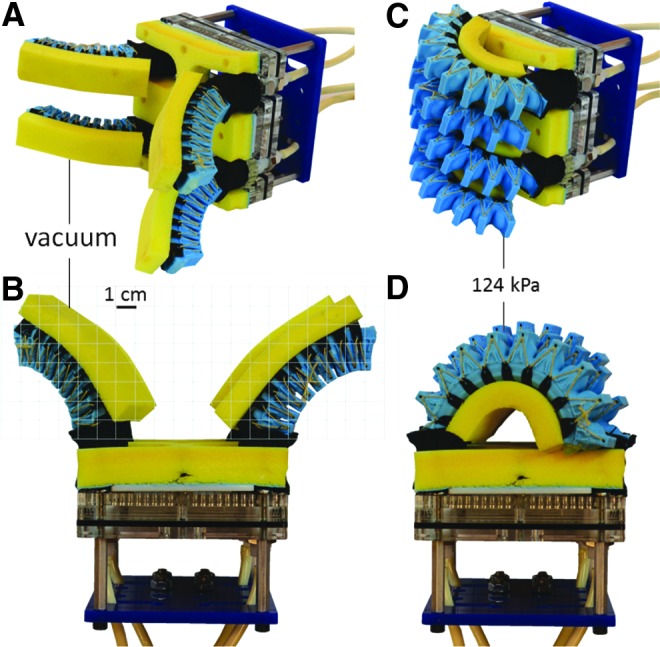
**(A, B)** Isometric and top view, respectively, of the bellows-type soft actuators under vacuum in the open pose state. The grid scale is approximated. **(C, D)** Isometric and top view of the bellows-type soft actuators pressurized to 124 kPa (18 psi). Color images available online at www.liebertpub.com/soro

Bellows-type soft actuators are a common architecture that creates asymmetric motion by unfolding the excess material in the bellows. Compared to fiber-reinforced actuators in which the elastomer material must strain to create motion, this unfolding approach places less strain on the material, which can increase the longevity of the actuator and lead to lower operating pressures. Bellows-type actuators also have the advantage that certain geometries can support bidirectional bending by alternating pressurized fluid and vacuum (Fig. 8). Furthermore, fiber reinforcements can be incorporated into bellows-type actuators to increase the actuator's operating pressure and output force, and to reduce its radius of curvature when actuated.

### Fabrication of a bellows-type soft actuator

One of the most challenging aspects of fabricating a bellows-type soft actuator is molding the inner geometry. The high aspect ratio features render any rigid core mechanically constrained and can lead to damage to the core and the actuator body during demolding. Mosadegh *et al.* used a two-step molding technique in which one-half of the bellow was molded and then bonded to the other half.^27^ However, this introduces a materially weak seam along the actuator's entire perimeter and becomes the source of most actuator failures. Marchese *et al.* present a method in which dissolvable cores are used to define the inner bellow geometry; however, this is a time-consuming process that requires a new core for each new actuator and time spent dissolving the core.^28^ To overcome these challenges, we developed a new fabrication method that uses reusable “soft cores” and a vacuum technique for rapid core removal.

**FIG. 9.**
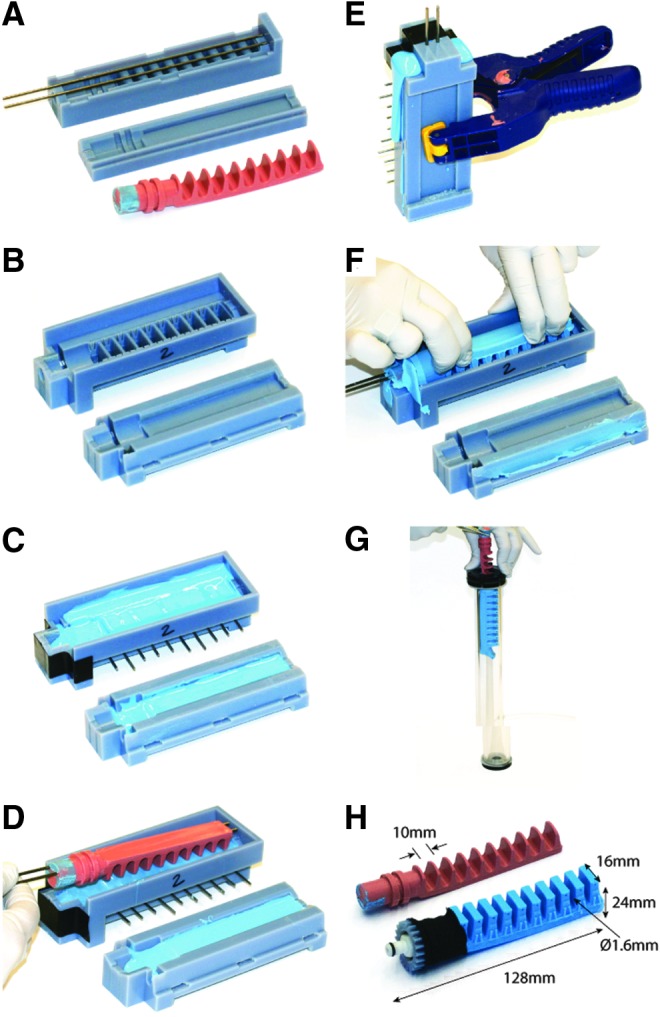
Schematic diagram outlining the stages of the soft, bellows-type actuator fabrication process. **(A)** A silicone form—a soft core—for the actuator's internal geometry is molded in 3D-printed molds. **(B)** 3D-printed molds define the actuator's exterior geometry. **(C)** Liquid polymer fills both halves of the mold. **(D)** The soft core form is positioned in the mold. **(E)** The two mold halves are clamped together and polymer is allowed to cure. **(F)** The actuator is removed from the mold as one piece. **(G)** The soft core is extracted with assistance from a vacuum tube. **(H)** Hardware for pneumatic coupling is installed. Color images available online at www.liebertpub.com/soro

The first step in fabricating a bellows-type soft actuator begins with molding the soft core, which will define the bladder geometry of the final actuator. Figure 9A depicts a silicone (M4601 by Wacker Chemical) soft core molded from a 3D-printed two-part mold. During this mold step, two metal rods were also co-molded with the core. These will be used to support and align the core in future mold steps. In the second step, a 3D-printed mold that defines the outer geometry of the soft actuator is prepared by applying mold release to all surfaces as well as to the soft core (Fig. 9B). Then the polymer (Smooth-Sil 950 by Smooth On) for the bellows-type actuator can be poured into both halves of the mold (Fig. 9C). Note that in this particular mold design, pins were added along the bottom of the mold to create through holes at the top of each bellow peak. These features serve as anchor points to add reinforcements, bridging materials, and other functions to the actuator. In the fourth step, the soft core is inserted into the mold, and rods are gently pushed into alignment slots built into the mold (Fig. 9D). The two halves of the mold are brought together and clamped (Fig. 9E). Once the polymer has cured, the part can be removed from the mold (Fig. 9F). In this example, the rods extend beyond the end of the actuator, which upon removal introduces two small holes at the end of the actuator. These can be plugged with a small amount of silicone glue (e.g., Sil-Poxy by Smooth On). In future work, we plan to eliminate this plugging step by cantilevering rods from one end of the mold to create an actuator with a closed end. In the seventh step, we demonstrate a novel application of vacuum pressure to remove the soft core from the actuator body (Fig. 9G). A flange molded at the open end of the actuator body is used to create an air-tight seal with the open end of a tubular vacuum chamber. When vacuum is applied, the actuator interior is subject to atmospheric pressure, whereas the actuator's outer surface is subject to vacuum. This creates a pressure differential that inflates and stabilizes the actuator body to allow the operator to pull out the core. Figure 9H depicts the undamaged, soft core removed from the actuator and ready for reuse. In the final assembly steps, the flange is cut off and a 3D-printed fitting is glued into the open end. Similar to the boa-type actuators, the fitting features gear teeth for integration with the gripper and integrated male quick disconnects for easy actuator installation and removal (Fig. 4).

### Gripper texture and other accessories

To expand the task versatility of the soft actuators, we developed a simple lacing technique to reversibly modify actuator textures. The advantage of this approach is that a wide variety of capabilities and functions can be added onto a small set of actuator types (i.e., the boa-type fiber-reinforced actuators and bellows-type actuators). The proposed lacing technique bonds different textures to a bridging material, which is then laced and tied to the actuator body. We chose ripstop nylon as a bridging material because it is flexible, laser-cut features are stable (i.e., no unraveling or fraying), and adhesives will bond to the surface. For the bellows-type actuator, which is molded without any fiber reinforcements, lacing has the added benefit of reinforcing the actuator and enabling it to operate at higher pressures (up to 170 kPa as opposed to 70 kPa without lace reinforcements).

In addition to supporting modular replacement of textures, the bridging material can support a variety of other functions, including acting as a connection interface for tools and instruments or bridging one actuator to another such as connecting a net between two actuators to improve grasping coverage.

**FIG. 10.**
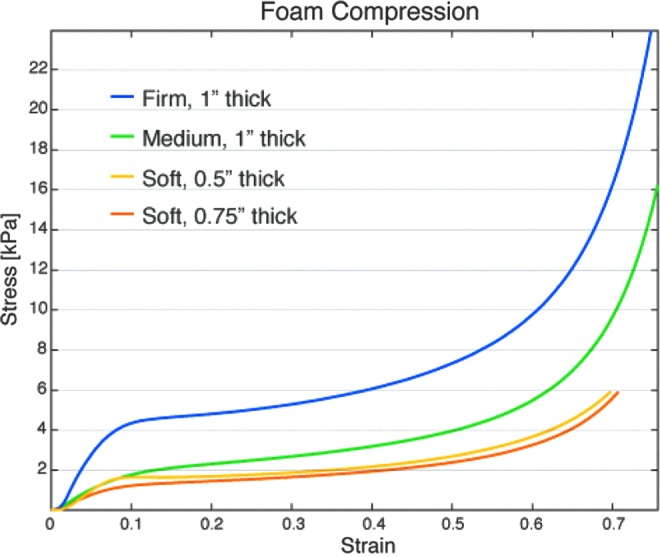
Stress–strain response of open-cell memory foam under compression. The nonlinear behavior supports a relatively consistent distribution of load for strains from 10% to 50%. Color images available online at www.liebertpub.com/soro

One material that has useful and unique properties is open-cell low-density memory foam—a lightweight and compliant material. In our observations, a half-inch (12.7 mm) layer of soft foam (part#: 86195K33; Mcmaster-Carr, Inc.) attached to bridging material does not significantly impede an actuator's range of motion. Furthermore, the foam offers mechanical advantages such as reducing the actuator's radius of curvature as it closes around an object, a useful feature for grabbing narrow specimens. Memory foam also has desirable nonlinear stress–strain properties that help distribute forces and conform to irregular shapes. Figure 10 captures this nonlinear behavior where several thicknesses and densities of open-cell memory foam were compressed in a materials characterization system (Instron 5544A). In all of the samples, the stress plateaus after ∼10% compressive strain. The softer foams exhibit the longest plateau where stress buildup is delayed until nearly 50% compression.

## Gripper Characterization

Since these gripper designs do not rely on sensors for force feedback, we empirically examined the contact pressure and resistive forces of the boa- and bellows-type actuators. This is important for biological material collection as well as underwater archeology, where understanding pressure distribution around an object and the gripper's load-carrying capacity is important before attempting recovery. It should be noted that the following results represent the capabilities of the actuators presented in this work and are meant to demonstrate the inherent safety, form closure, and grasp strength of the chosen designs. A gripper's performance under these metrics can be adjusted easily through changes in geometry and materials.

### Contact pressure

**FIG. 11.**
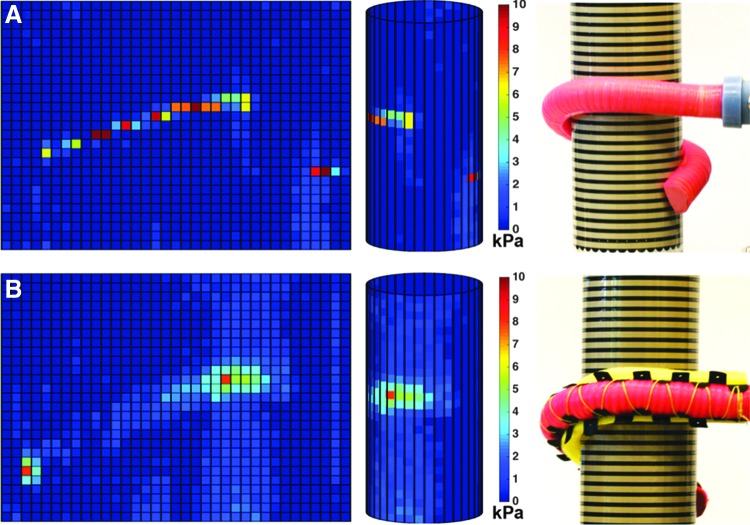
Pressure map of a boa-type fiber-reinforced soft actuator—without **(A)** and with **(B)** memory foam liner—wrapping around a 2 inch diameter cylinder with a Tekscan pressure map sensor. Both configurations apply relatively low contact forces (<10 kPa) to the cylinder; however, the memory foam liner **(B)** improves pressure distribution and reduces peak forces. Color images available online at www.liebertpub.com/soro

Several bench-top experiments were performed to understand the magnitude of the force and pressure applied to static objects. A 63.5-mm diameter plastic tube was selected as the target object because both soft actuator types could cradle more than half the circumference of the tube and the radius of curvature was suitable for wrapping with a Tekscan (model: 5051-20) pressure mapping sheet (Fig. 11). Several actuator configurations were evaluated including the boa- and bellows-type actuators with and without memory foam textures. Figure 11 depicts the experimental setup and corresponding pressure map for the boa-type actuator. Pressurized to 310 kPa (45 psi), the boa-type actuator without a foam texture had a narrow pressure distribution with most pressures ranging from 6 to 10 kPa, whereas the boa-type actuator with a foam inner surface had a greater area of contact with most recorded contact pressures below 5 kPa (0.72 psi). The bellows-type actuators (pressurized to 69 kPa [10 psi]) had a similar response in which the maximum pressures detected were 2 kPa (0.29 psi) and 7 kPa (1 psi), without and with foam, respectively.

### Pull force

Grip strength was evaluated with a materials characterization system (model: 5544A single column; Instron) that analyzed load–extension characteristics for several gripper scenarios, including load direction (i.e., vertical vs. horizontal) and object size. In the experimental protocol, an acrylic tube ranging in diameter from 12.7, 25.4, and 50.8 mm was positioned at the palm of the gripper, and the actuators were inflated to their target pressures to enclose the cylinder. All bellows-type actuators were pressurized to 124 kPa (18 psi) and boa-type actuators were inflated to 310 kPa (45 psi) with one exception being the boa-type actuator that was pressurized to 345 kPa (50 psi) to fully close around the 12.7 mm diameter tube. Furthermore, the grip strength of the bellows-type actuators was evaluated with only two actuators, one opposing the other. The gripper was anchored to the table and the Instron was pulled on the tube at a fixed velocity (8 mm/s) until the cylinder was pulled from the actuator's grasp. Each test configuration was evaluated five times and the results were averaged. In the following plotted results, the solid black line represents the average of the results whereas the shaded area represents one standard deviation.

**FIG. 12.**
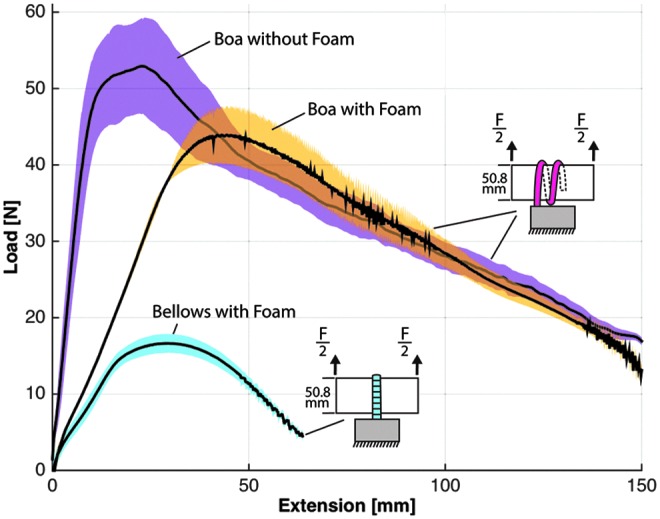
Vertical load–extension response of the boa-type gripper with and without a foam inner surface and the bellows-type gripper with foam gripping a 50.8-mm diameter. Color images available online at www.liebertpub.com/soro

**FIG. 13.**
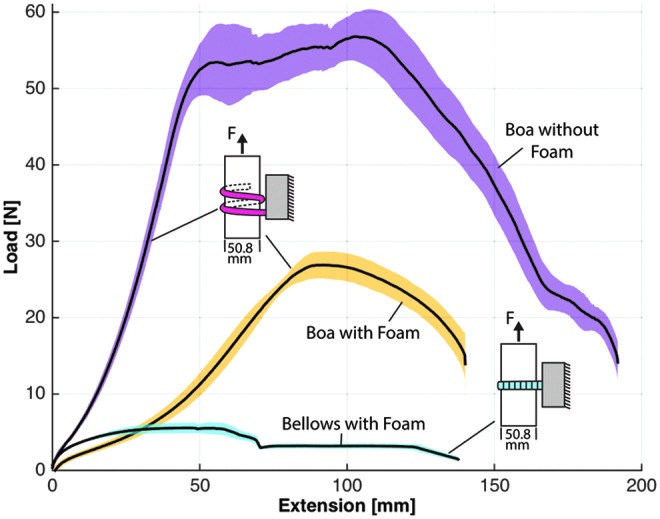
Horizontal load–extension response of the boa-type gripper with and without foam and the bellows-type actuators with foam gripping a 50.8-mm diameter acrylic tube. Color images available online at www.liebertpub.com/soro

**FIG. 14.**
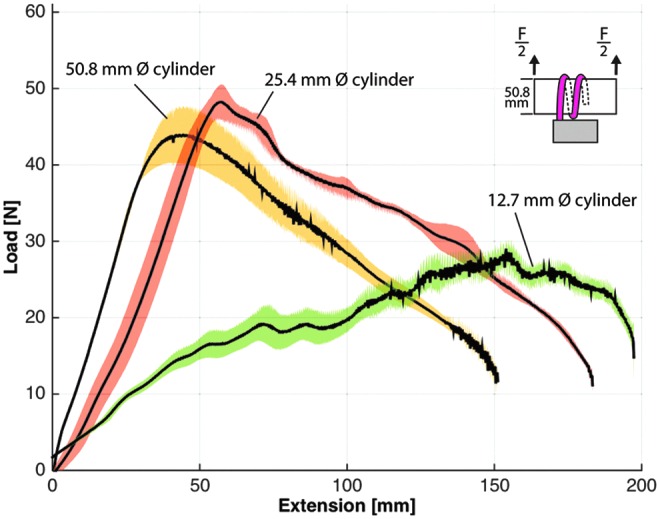
Vertical load–extension response of the boa-type gripper with foam for three different diameter acrylic tubes—12.7, 25.4, and 50.8 mm diameter. Color images available online at www.liebertpub.com/soro

While gripping the 50.8 mm diameter tube, the bellows-type gripper had the greatest resistive force in the vertical direction (16.6 N) (Fig. 12), but offered relatively little resistive force in the horizontal direction (5.6 N) (Fig. 13). The boa-type gripper had significantly higher vertical and horizontal resistive forces across a range of tube diameters. In vertical pull tests with the 50.8 mm diameter tube, the boa-type gripper without foam had an average maximum holding force of 52.9 N, whereas peak average force of the actuator with foam was 44 N (Fig. 12). Furthermore, in experiments in which the tube diameter was reduced to 25.4 and 12.7 mm, the holding force remained high compared to the bellows-type actuator (Fig. 14).

**FIG. 15.**
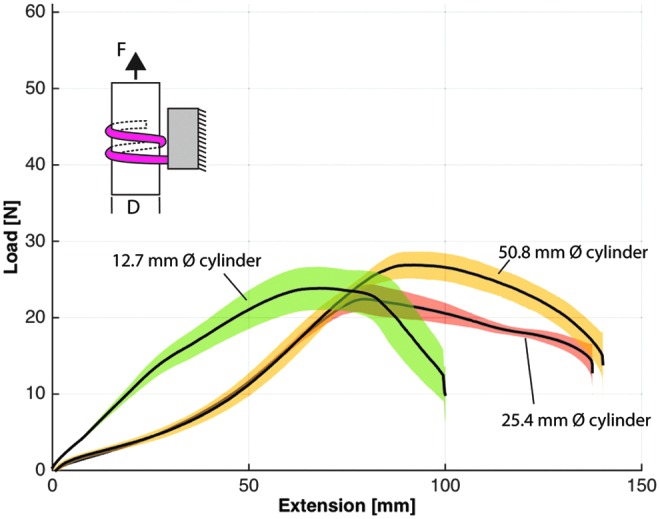
Horizontal load–extension response of the boa-type gripper with foam for three different diameter acrylic tubes—12.7, 25.4, and 50.8 mm diameter. Color images available online at www.liebertpub.com/soro

In horizontal pull tests, the boa-type gripper actuator without foam had higher resistive forces (peak average of 56.8 N), whereas the resistive force of the actuator with foam was approximately half (approximate peak average of 26.9 N) (Fig. 13). This is likely because of the differences in friction coefficients between silicone rubber and foam on the acrylic tube. When the tube diameter was varied for the boa-type gripper with foam, the peak average resistive forces were similar and were 23.8 N for 12.7 mm and 22.4 N for 25.4 mm diameter tubes (Fig. 15).

## Pilot Study

**FIG. 16.**
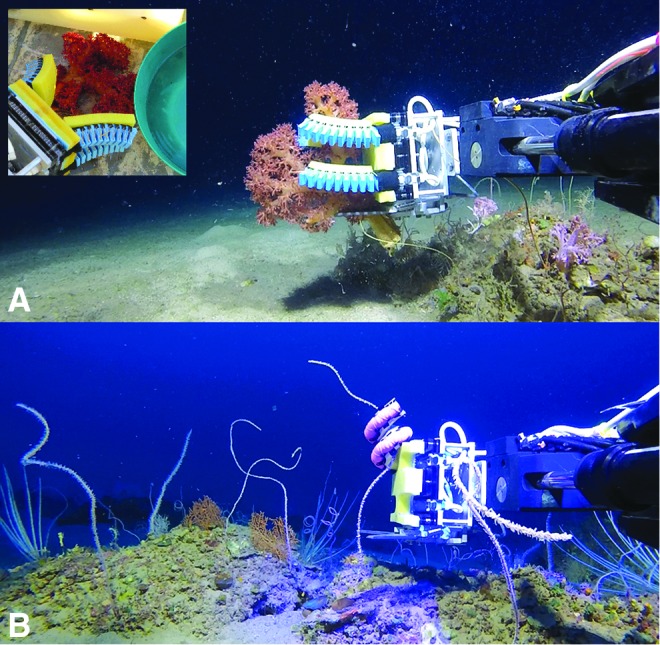
**(A)** Bellows-type gripper collecting soft coral (*Dendronephthya sp*.) with *inset* image showing the sample on the deck of the ship. **(B)** Boa-type gripper collecting an Alcyonacean whip coral at a depth of 100 m. The arm and gripper were visually controlled using the Deep Reef's onboard cameras. Color images available online at www.liebertpub.com/soro

In a small pilot study, we brought the Deep Reef ROV and soft robotic grippers to the Gulf of Eilat in the northern Red Sea. To our knowledge, these field trials were the first to demonstrate deep sea marine biology collection and manipulation with soft robotic grippers. Figure 16 presents the two gripper configurations that were evaluated: one with two opposing pairs of bellows-type actuators (Fig. 16A) and the other with a single boa-type actuator (Fig. 16B). Both gripper configurations were operated from a single hydraulic source and had memory foam lining on the actuators and the palm. Using the bellows-type gripper we retrieved a red soft coral (*Dendronephthya sp.*) after landing the ROV on the sea floor. The four bellows-type actuators gently closed around the specimen without damaging any of the branches. The boa-type gripper proved very effective at wrapping around long and narrow (<12 mm diameter) coral whips that extended vertically from the sea floor. The large effective range of motion of the boa-type actuator combined with the coiling effect reduced the burden on the operator to position the ROV and gripper in the optimal position. We repeatedly demonstrated that once a specimen was in reach, the boa-type actuator could draw it in and close around it. This is an important capability because the seafloor is not always suitable for landing the ROV (e.g., rough terrain, risk of stirring up sediment, or tangle hazards such as fishnets may be nearby). Consequently, the operator must try to maintain a stable hover under external influences (i.e., currents and tether tensioning) and slowly approach the target. Therefore, arm and gripper designs that extend reach and quickly grasp can improve sample recovery success while minimizing damage to the sample and its surroundings.

In this pilot study, the soft gripper was deployed in more than a dozen dives at depths ranging from 100 to 170 m. On most dives, the goal was to demonstrate that the gripper could safely grab a specimen without removal. However, to evaluate the sample recovery process from start to finish, two species, which are abundant in the Red Sea, were recovered (see the inset image of the recovered red soft coral in Fig. 16A). Between dives we were able to verify the versatility of the gripper design; the change from one gripper configuration to another (including purging the hydraulic lines of air bubbles) could be completed in <5 min.

## Conclusions and Future Work

We presented new advancements in the design, fabrication, and field testing of soft robotic grippers for deep sea collection of fragile biological specimens. Specifically, we describe two different soft material actuator designs—boa-type fiber-reinforced actuators and bellows-type actuators—that can gently conform around objects with the control input of a single hydraulic line. Furthermore, we describe a new fabrication technique for the rapid production of bellows-type actuators using soft cores and a vacuum-assisted demolding step. We also describe a method of adding bridging material to soft actuators to change an actuators radius of curvature, texture, and contact forces. Lastly, the short field trial showed that the instrument performs as expected and can offer a game-changing approach to how deep sea organisms are collected in the future.

This work represents the first step of a larger vision to create haptic controlled soft robotic “arms” and “fingers” that can be as functional as (or more functional than) a human SCUBA diver. We envision the capability of performing complex scientific experiments and collection by an ROV or submersibles. This could include delicate collection, manipulation, or *in situ* measurement of deep reef organisms. We also envision that this can be combined with the capability of applying RNA stabilizers, such as RNAlater, to samples to facilitate gene expression and transcriptomic analysis.^29,30^ Alternatively, waiting to stabilize RNA until after the sample arrives at the surface results in the sample being exposed to drastic changes in temperature, pressure, and light. This can stress the organism and lead to a gene profile that includes upregulation of stress responses. The capability of performing these tasks *in situ* opens up a vast potential to the marine biological and molecular biological community as we move forward with our understanding of physiology and genomics of deep reef and deep sea organisms.

## Figures and Tables

**APPENDIX FIG. A1.**
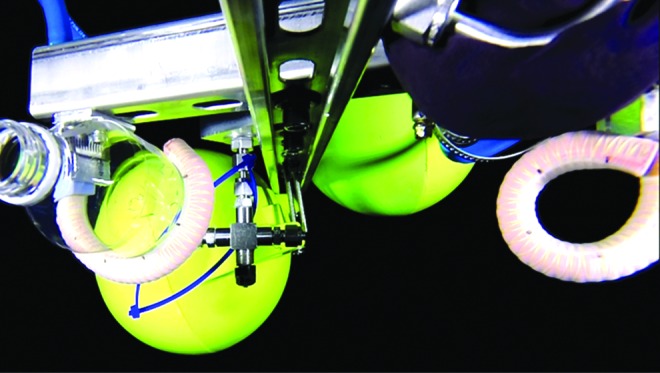
A test rig lowered more than 800 m to evaluate the influence of high-hydrostatic forces on the soft fiber-reinforced actuators. Color images available online at www.liebertpub.com/soro

## Notes

^*^ Falcon ROV by Seaeye, www.seaeye.com/falcon.html

^†^ HLK-43000, www.hydro-lek.com/datasheets/hlk43000a.pdf

## Appendix

At the start of the study, it was unclear how the soft actuators (i.e., the materials, the fiber reinforcements, the fittings, etc.) would perform under high-hydrostatic pressure. To stress test the actuators, a simple rig was assembled that included mounts for two actuators, two LED lamps, and a video camera rated for a depth of 1000 m. A fixed volume of water (i.e., constant fluid mass) was pumped into the actuators to cause them to bend and the outlet was locked. The entire rig was lowered to a depth of more than 800 m—a hydrostatic pressure more than 8000 kPa (1170 psi). Video of the descent and ascent revealed no noticeable impact on the actuator's structural integrity or its ability to hold a curled state (Appendix Fig. A1).
